# Supplementary material for: MetaRibo-Seq measures translation in microbiomes
Source: Nat Commun. 2020 Jun 29;11:3268. doi: 10.1038/s41467-020-17081-z (PMC7324362; doi:10.1038/s41467-020-17081-z)
Supplement: Supplementary file 10 — Supplementary Data 7 [file 41467_2020_17081_MOESM10_ESM.zip › File2/Confidence_High_Taxonomy/52497_out.krona.html]

Javascript must be enabled to view this page.

members
magnitude
magnitudeUnassigned
count
unassigned
taxon
rank

52497\_out

1

1
superkingdom
2759

4751
kingdom
1

1
subkingdom
451864

4890
1
phylum

147538
subphylum
1

1
class
147545

451871
subclass
1

5042
1
order

family
1
1131624

1
genus
5086

5088
species

SRS144506\_contig\_number\_contig-100\_25737.25737
1
